# From the Beginning of the Korean Gynecologic Oncology Group to the Present and Next Steps

**DOI:** 10.3390/cancers16193422

**Published:** 2024-10-09

**Authors:** Kyung-Jin Min, Nam Kyeong Kim, Jae-Yun Song, Min Chul Choi, Shin Wha Lee, Keun Ho Lee, Min Kyu Kim, Sokbom Kang, Chel Hun Choi, Jeong-Won Lee, Eun-Ju Lee, Keun-Yong Eom, Sang Wun Kim, Hanbyoul Cho, Sun Joo Lee, Myong Cheol Lim, Jaeman Bae, Chong Woo Yoo, Kidong Kim, Dae-Yeon Kim, Chulmin Lee, Sang Young Ryu, Seob Jeon, Jae-Weon Kim, Byung-Ho Nam, Soon-Beom Kang, Kyung Tae Kim, Joo-Hyun Nam, Byoung-Gie Kim, Yong-Man Kim, Jae-Hoon Kim

**Affiliations:** 1 Department of Obstetrics and Gynecology, Korea University Ansan Hospital, Ansan 15355, Republic of Korea; mikji@naver.com (K.-J.M.); 54305knk@gmail.com (N.K.K.); 2 Department of Obstetrics and Gynecology, Korea University Anam Hospital, Seoul 02841, Republic of Korea; sjyuni105@gmail.com; 3 Comprehensive Gynecologic Cancer Center, CHA Bundnang Medical Center, Seongnam 13496, Republic of Korea; oursk79@cha.ac.kr; 4 Department of Obstetrics and Gynecology, Asan Medical Center, Seoul 05505, Republic of Korea; swhlee@amc.seoul.kr (S.W.L.); kdyogt@gmail.com (D.-Y.K.); amcymkim@gmail.com (Y.-M.K.); 5 Department of Obstetrics and Gynecology, Seoul St. Mary’s Hospital, Seoul 06591, Republic of Korea; hohoho@catholic.ac.kr; 6 Division of Gynecologic Oncology, Department of Obstetrics and Gynecology, Samsung Changwon Hospital, Sungkyunkwan University School of Medicine, Changwon 51353, Republic of Korea; minkyukim@skku.edu; 7 Center for Gynecologic Cancer, National Cancer Center, Goyang 10408, Republic of Korea; sokbom@ncc.re.kr (S.K.); mclim@ncc.re.kr (M.C.L.); 8 Department of Obstetrics and Gynecology, Samsung Medical Center, Seoul 06351, Republic of Korea; huna0@naver.com (C.H.C.); garden.lee@samsung.com (J.-W.L.); bgkim@skku.edu (B.-G.K.); 9 Department of Obstetrics and Gynecology, Chungang University Hospital, Seoul 06973, Republic of Korea; ejlee@cau.ac.kr; 10 Department of Radiation Oncology, Seoul National University Bundang Hospital, Seongnam 13620, Republic of Korea; 978sarang@hanmail.net; 11 Department of Obstetrics and Gynecology, Yonsei Cancer Center, Seoul 03722, Republic of Korea; san1@yuhs.ac; 12 Department of Obstetrics and Gynecology, Yonsei University Gangnam Severance Hospital, Seoul 06273, Republic of Korea; hanbyoul@yuhs.ac; 13 Department of Obstetrics and Gynecology, Konkuk University Medical Center, Seoul 05030, Republic of Korea; lsj1121@yahoo.co.kr; 14 Department of Obstetrics and Gynecology, Hanyang University Seoul Hospital, Seoul 04763, Republic of Korea; obgybae@hanyang.ac.kr; 15 Department of Pathology, National Cancer Center, Goyang 10408, Republic of Korea; cwy@ncc.re.kr; 16 Department of Obstetrics and Gynecology, Seoul National University Bundang Hospital, Seongnam 13620, Republic of Korea; kidong.kim.md@gmail.com; 17 Department of Obstetrics and Gynecology, Cha University Ilsan Medical Center, Goyang 10414, Republic of Korea; morula3@gmail.com; 18 Department of Uterine and Ovarian Cancer Center, Korea Cancer Center Hospital, Seoul 01812, Republic of Korea; ryu@kcch.re.kr; 19 Department of Obstetrics and Gynecology, Soonchunhyang University Hospital Cheonan, Cheonan 31151, Republic of Korea; sjeon@schmc.ac.kr; 20 Department of Obstetrics and Gynecology, Seoul National University Hospital, Seoul 03080, Republic of Korea; kjwksh@snu.ac.kr; 21 Herings, Seoul 06144, Republic of Korea; byunghonam@heringsglobal.com; 22 Department of Obstetrics and Gynecology, Hosan Women’s Hospital, Seoul 06023, Republic of Korea; ksboo308@gmail.com; 23 Geumsan Geriatric Hospital, Geumsan 32753, Republic of Korea; kimkt0103@gmail.com; 24 Asan Medical Center, University of Ulsan College of Medicine, Seoul 05505, Republic of Korea; jhnam@amc.seoul.kr

**Keywords:** Korean Gynecologic Oncology Group, gynecologic cancer, clinical trial

## Abstract

**Simple Summary:**

Established in 2002, the Korean Gynecologic Oncology Group (KGOG) has presented improved clinical outcomes based on multi-center clinical trials. To date, KGOG has approved 156 studies and published 68 KGOG-led studies. The organization aims to advance gynecologic cancer research through sustained efforts and international collaboration.

**Abstract:**

The Korean Gynecologic Oncology Group (KGOG) was established in 2002 and is the only organization in Korea conducting multi-center clinical trials for gynecologic cancers. Since its re-establishment as a non-profit organization in 2021, KGOG has grown significantly, now including 207 gynecologic oncology specialists from 76 hospitals. This growth is a testament to the dedication and hard work of all those involved in the organization. KGOG is committed to maximizing the activation of multi-center clinical research through policies that support patients with rare diseases and gynecologic cancer research, focusing on strengthening institutional capacity, equalizing participation opportunities, and enhancing information sharing. A significant milestone for KGOG was becoming a member of the US Gynecologic Oncology Group (GOG) in 2005, allowing participation in GOG clinical trials. KGOG later joined the Gynecologic Cancer InterGroup (GCIG) and strengthened its capabilities by hosting the first Endometrial Cancer Consensus Conference—Clinical Research (ECCC-CR) in 2023. KGOG holds biannual meetings and symposia, as well as 224 operating committee meetings annually to review the discussions of the Tumor Site Committee. KGOG has conducted 156 investigator-initiated trial (IIT) or sponsor-initiated trial (SIT) studies as KGOG-led or participated in research. Currently, 18 studies are registered, and 10 are in preparation. To date, 68 papers have been published. KGOG conducts six national projects and collaborates with external organizations such as the NRG Oncology Foundation, Gynecologic Oncology Group Partners (GOG-P), GCIG, East Asian Gynecologic Oncology Trial group (EAGOT), and the Japanese Gynecologic Oncology Group (JGOG). Through collaboration with renowned international research institutions, KGOG has significantly expanded the scope of its research, achieving noteworthy clinical outcomes. This report not only introduces the history and recent status of KGOG but also presents the exciting future direction of the organization, filled with potential breakthroughs and advancements in gynecologic oncology research.

## 1. Introduction

In the fall of 2002, the KGOG was established by leading figures in the field of gynecologic oncology in Korea, with the support of the Korean Society of Gynecologic Oncology (KSGO). This initiative aimed to model itself after advanced multi-institutional clinical trial organizations, such as the Gynecologic Oncology Group (GOG) in the United States, to conduct multi-institutional clinical trials. Over the past 20 years, KGOG has built upon the successes and failures of these trials to establish itself as a successful clinical research organization, continuing its efforts to make significant contributions to the field.

This paper aims to present KGOG’s history and research achievements and propose strategies and directions for its future development.

## 2. History of KGOG

### 2.1. The Beginning of KGOG

In October 2002, during the 9th International Gynecologic Cancer Society (IGCS) Meeting held in Seoul concurrently with the Korea–Japan Gynecologic Cancer Joint Meeting (KJGCJM), several professors from the KSGO recognized the need for a clinical research organization to lead clinical trials in gynecologic oncology in Korea. They made multifaceted efforts to overcome the surrounding opposition to data sharing and the immature era of clinical research. Consequently, the KGOG was established in October 2002 as an organization dedicated to clinical trials for gynecologic cancers in Korea [1,2].

In January 2003, the first operating committee meeting was held, where the KGOG preparatory committee was formed, drafting the KGOG bylaws, and discussions on clinical trial protocols began. Based on this, the first research committee was convened. The first KGOG workshop was held in August 2005. KGOG holds biannual meetings (spring and autumn) and two symposiums/workshops annually. In its initial stages, KGOG comprised the president, research committee, and operating committee, with advisory subcommittees for cervical cancer, ovarian cancer, endometrial cancer, pathology, radiation therapy, medical oncology, and translational research.

### 2.2. Research Achievements of KGOG

The classification number of the KGOG protocols was determined by organ to be a four-digit number starting with 1 for the cervix (KGOG 1XXX), 2 for the uterine body (KGOG 2XXX), and 3 for the ovary (KGOG 3XXX). The protocol for Surgery and Developmental Diagnostic and Therapeutics (DDT) was classified as 4 (KGOG 4XXX). In August 2004, the first KGOG protocol, KGOG 1001 (A Phase II Trial of Radiation Therapy with Concurrent Paclitaxel/Carboplatin Chemotherapy in High-risk Cervical Cancer Patients after Radical Hysterectomy), was initiated, and the study was conducted until 2010. The study was published in June 2013 [3]. Subsequently, the KGOG 3001 (An Open label, Single arm and Multi-center Phase II Clinical Trial of Gemcitabine Triplet [Paclitaxel + Carboplatin + Gemcitabine] as Consolidation Chemotherapy in Patients with Advanced Epithelial Ovarian Cancer) and KGOG 2001 (A Phase II Trial of Radiation Therapy with Concurrent Paclitaxel Chemotherapy in High-risk Endometrial Cancer Patients after Operation) protocols began in July and August 2005, respectively. The KGOG 2001 study was published in September 2014 [4]. The first study published by the Ovary–Fallopian tube Tumor Site Committees was KGOG 3003, a retrospective study of clear cell carcinoma of the ovary [5]. To date, 156 KGOG protocols have been developed and carried out. KGOG-led and -participated clinical trials are summarized in Table 1. Among them, the list of studies conducted and published by researchers affiliated with KGOG over the past five years is summarized in Table 2.

### 2.3. Interaction with Other International Research Organizations

Since its establishment, the KGOG has collaborated with prominent international institutions leading multi-center research, such as the GOG Legacy and NRG in the United States, European Network for Gynaecological Oncological Trial group (ENGOT) in Europe, and the Gynecologic Cancer InterGroup (GCIG). As a member of these collaborative research efforts, KGOG has significantly contributed to international joint studies. KGOG became an associate member of the GOG in July 2005. After the KGOG-NCI-US Embassy Cooperation Meeting in April 2007, KGOG signed an agreement with GOG to conduct joint research and clinical trials. The first KGOG meeting was held in January 2008 during the GOG semi-annual meeting in San Diego, USA. Since then, KGOG has been participating in the GOG meetings twice a year as a full member and continues to cooperate closely with NRG, the successor of GOG.

KGOG became a full member of GCIG in October 2007 and has since participated in numerous studies. In November 2023, KGOG hosted the first Endometrial Cancer Consensus Conference—Clinical Research (ECCC-CR) in Songdo, Korea, alongside the KGOG semi-annual meeting, focusing on clinical trial guidelines for endometrial cancer [30].

Additionally, KGOG has maintained early interactions with Japan through the Japanese Gynecologic Oncology Group (JGOG) and KJGCJM. The first KGOG–JGOG collaboration meeting was held in April 2016. Since then, KGOG and JGOG have developed an advanced cooperative relationship, holding biannual face-to-face meetings to develop joint protocols [31]. Since 2012, KGOG has also been holding regular meetings with the Shanghai Gynecologic Oncology Group (SGOG) through the KGOG–SGOG meetings [32].

Recently, KGOG, along with JGOG, the Chinese Gynecological Cancer Society (CGCS), and the Taiwan Gynecologic Oncology Group (TGOG), established the East Asian Gynecologic Oncology Trial Group (EAGOT) in November 2021. EAGOT aims to develop clinical trial protocols for gynecologic cancers in East Asian women and expand research exchange among East Asian countries, with KGOG leading these efforts [33,34].

## 3. Current KGOG

### 3.1. Mission and Objectives of KGOG

The mission of the KGOG is three-fold as follows: First, to advance gynecologic oncology through collaborative multi-institutional research. This involves conducting and supporting domestic and international multi-center clinical trials related to the diagnosis, surgery, chemotherapy, and radiotherapy of gynecologic cancers. Second, to contribute to public health by developing new chemotherapy protocols and surgical techniques. This goal is furthered through the education and training of clinical trial experts in gynecologic cancer, enhancing the overall quality of care and treatment outcomes. Third, rational clinical trial policies should be proposed. By engaging in policy research, KGOG aims to shape effective clinical trial policies that reflect the latest advancements and best practices in gynecologic oncology. To achieve its mission, KGOG undertakes the following activities: (1) It develops, conducts, and exchanges information on multicenter clinical trial protocols for gynecologic cancer, ensuring standardized and collaborative research efforts across institutions. (2) It also participates in policy research to formulate and refine clinical trial policies, ensuring they are evidence-based and effective. (3) It is strengthening cooperation and exchanges with domestic and international institutions related to gynecologic cancer research. This fosters a global network of collaboration and information sharing. (4) Educating and training experts involved in gynecologic cancer research. This commitment ensures high expertise and competence among clinical trial professionals. (5) We promote and enlighten gynecologic cancer research to raise awareness and encourage investment in this critical field. (6) This engages in other activities deemed necessary by KGOG to fulfill its mission and achieve its objectives.

### 3.2. Structure of KGOG

**Figure 1:**
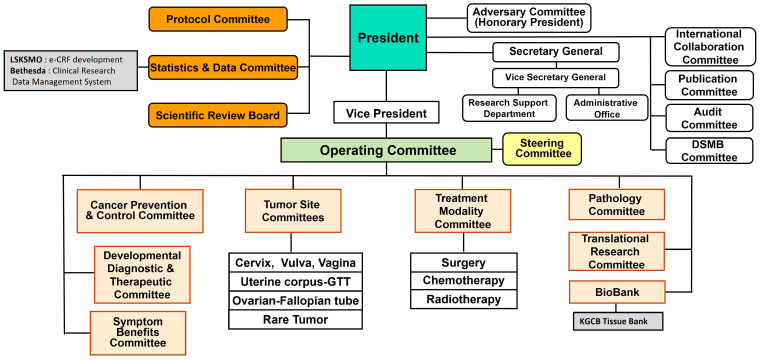
Structure of KGOG.

KGOG has expanded to its current structure over approximately 20 years, passing through the leadership of five presidents: Professor Soon-Beom Kang (2002–2010), Professor Joo-Hyun Nam (2010–2014), Professor Byung-Gie Kim (2014–2016), Professor Yong-Man Kim (2016–2021), and Professor Jae-Hoon Kim (2021–present). The current organizational chart of KGOG is shown in Figure 1. In 2024, KGOG consisted of 4 Tumor Site Committees (Cervix, Vulva, Vagina; Uterine Corpus-GTT; Ovary–Fallopian tube; Rare Tumor), 3 Treatment Modality Committees (Surgery; Chemotherapy; Radiotherapy), and 14 other committees (Advisory; Operating; Steering; Protocol; Statistics and Data; International Collaboration; Publication; Audit; DSMB; Cancer Prevention and Control; Developmental Diagnostic and Therapeutic; Symptom Benefits; Pathology; Translational Research), and 2 supportive departments (SRB and Biobank). The operating Committee reviews the progress of KGOG research every month, while the Steering Committee is an ad hoc committee that primarily handles financial and administrative matters.

### 3.3. Development Process of New Protocols

**Figure 2:**
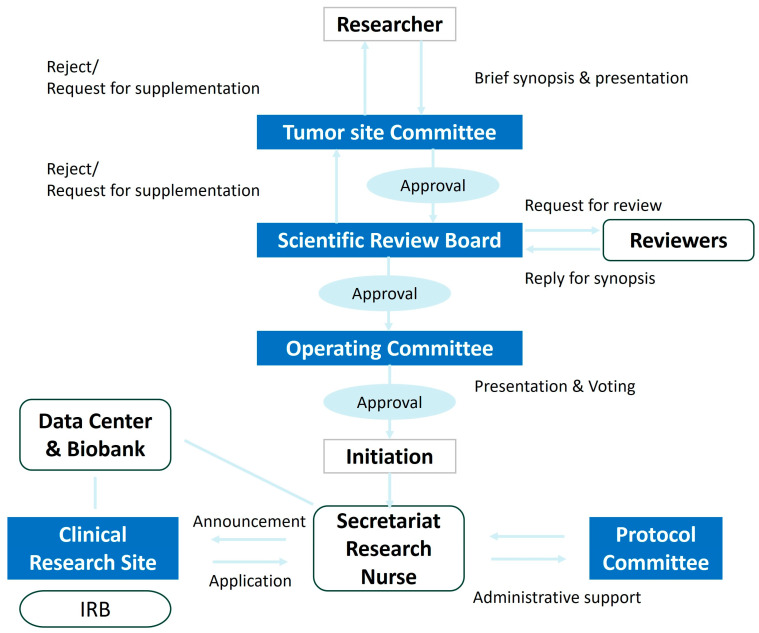
Flow-chart of development of a new protocol in KGOG.

The researcher submits a summary-formatted research proposal to the Tumor Site Committee for initial review. If the study is deemed to have clinical value and is suitable for KGOG, the Tumor Site Committee chair submits the proposal to the Scientific Review Board (SRB) for a detailed secondary review. Comprising five internal and external reviewers plus one chair, the SRB assesses the rationality, feasibility, and ethical considerations of all clinical trials to be conducted by KGOG. Upon SRB approval, the principal investigator will present the proposal to the Operating Committee based on the discussions that took place within the Tumor Site Committee and SRB, and the Operating Committee attendees will discuss whether to proceed with the study. If more than two-thirds of attendees agree during the second review, the study is approved as a KGOG protocol. If not approved, decisions may include re-review, deferral, or rejection. Once the Protocol Committee approves the full protocol, the principal investigator drafts a research contract with KGOG and obtains a research number through the KGOG Secretariat to recruit participating institutions. The development process of new protocols is detailed in Figure 2.

As of January 2024, KGOG is composed of a comprehensive national network of 76 institutions and hospitals, with a total of 207 gynecological oncologists, statisticians, radiation oncologists, pathologists, and medical oncologists as members (Supplementary Figure S1). In proportion to the population and number of patients, most hospitals are located in the metropolitan area, but for this reason, patients have high access to hospitals and can be centrally concentrated, so KGOG covers not only large-scale studies such as Phase III but also small-scale studies. The clinical trials actively conducted by KGOG in 2024 are summarized in Table 3.

### 3.4. Partnership between GOG-P and KGOG

**Figure 3:**
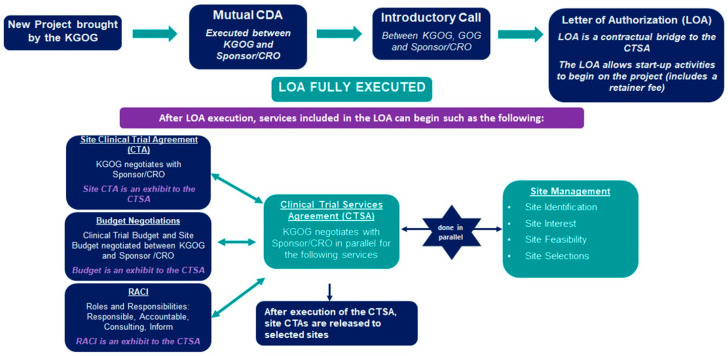
New study initiation process between GOG-P and KGOG. CDA: Confidential Disclosure Agreement; CRO: Contract Research Organization; CTSA: Clinical Trial Services Agreement; GOG: Gynecologic Oncology Group; KGOG: Korean Gynecologic Oncology Group; RACI: Responsible, Accountable, Consulting, Inform.

In 2014, GOG integrated with the National Surgical Adjuvant Breast and Bowel Project (NSABP) and the Radiation Therapy Oncology Group (RTOG) to form NRG Oncology. As a result, since 2011, Phase 3 studies on gynecologic oncology have significantly decreased in the United States, and KGOG has faced many limitations in newly participating in NRG studies since the mid-2010s. To overcome these challenges, GOG established the GOG Foundation in 2017, consisting of GOG Partners and the NRG Oncology gynecologic program [35]. After much discussion, the KGOG–GOG collaboration agreement was signed in October 2023, allowing KGOG to participate in GOG Partners studies. This has expanded opportunities for KGOG to participate in major international clinical trials, focusing on SIT. The new study initiation process is displayed in Figure 3, with a legal review conducted by both institutions.

Upon receiving the Letter of Authorization (LOA), start-up activities for the project begin, acting as a contractual bridge to the CTSA (Clinical Trial Site Agreement). After LOA execution, negotiations for the Site Clinical Trial Agreement (CTA), budget, and RACI (Roles and Responsibilities) are conducted in parallel with KGOG and the sponsor/CRO through the CTSA. Currently, five study protocols are in the new study initiation process. By establishing a partnership with GOG Partners, information about Asian gynecologic cancer patients will be reflected in new drug information, enabling the selection of more appropriate treatment options. Furthermore, through continuous development, KGOG will be able to keep pace with global trends in the field of gynecologic cancer clinical research.

The KGOG is dedicated to advancing gynecologic cancer research and treatment through core services such as site selection assistance, financial management, investigator meetings, and effective communication. These services ensure efficient study execution, transparent financial transactions, and robust collaboration among researchers. KGOG aims to transform the standard of care in gynecologic oncology through close collaboration with researchers and research organizations both in Korea and worldwide. We are committed to advancing investigator-initiated trials based on national grants and strengthening our partnership with research organizations, led by GOG-P to expand sponsor-initiated studies. By scientifically conducting clinical trials proposed by individual researchers or sponsors, we aim to elevate the standards of gynecologic cancer care.

### 3.5. Challenges of KGOG in the Future

Over the past 20 years, KGOG has achieved many accomplishments in the field of clinical trials and treatments for gynecologic cancers. However, it is true that there are still several challenges to overcome. First, while the government is streamlining regulatory processes to stimulate the medical market, it has not yet fully met the enthusiasm of researchers and patients for clinical trials. Second, although the patient recruitment rate is higher in Korea compared to the U.S. or Western Europe where clinical trials are more active, the relatively smaller population in Korea can make it difficult for multi-national pharmaceutical companies or contract research organizations (CROs) to prioritize Korea for large-scale, multi-center trials. Lastly, since Korea is a non-English-speaking country, language and cultural barriers must be addressed. All clinical trial documents, including patient consent forms, must be translated into Korean, and professional translators need to be hired to ensure the accuracy and quality of these translations. Therefore, to successfully conduct clinical research in Korea, it is crucial to gain knowledge about the factors that influence clinical research and the methods that can be used to overcome these challenges.

## 4. Conclusions

As mentioned above, KGOG has grown and developed significantly on its own and with international exchanges over the past 20 years. In the future, KGOG plans to develop KGOG-led investigator-initiated trials in quantity and quality to lead the clinical trials for new drug discovery and development, which have been rapidly growing recently. Additionally, based on solid relationships with international research organizations, KGOG will establish “Global Standards” for gynecologic oncology research and strive to promote gynecologic cancer research beyond East Asia to a global scale through exchanges with GOG-P, ENGOT, and EAGOT.

## Figures and Tables

**Table 1:** Summary of KGOG-led and -participated clinical trials.

	KGOG-Led	KGOG-Participated	Total
No. of protocols	119	37	156
No. of prospective trials	76 *	37	113
No. of retrospective trials	43	0	43
Committee			
Cervix, Vulva, Vagina	37	11	48
Uterine Corpus-GTT	25	7	32
Ovary–Fallopian tube	45	19	64
Surgery, DDT	12	0	12

* Two survey studies were included. DDT: Developmental Diagnostic and Therapeutics; KGOG: Korean Gynecologic Oncology Group; GTT: Gestational Trophoblastic tumor.

**Table 2:** Summary of studies conducted and published by KGOG.

Year	Author	Cancer Type	Study Type	KGOG-Led/Participated
2020	MC Choi et al. [6]	Ovarian Cancer	Retrospective	KGOG-led
	MK Kim et al. [7]	Endometrial Cancer	Retrospective	KGOG-led
	ES Paik et al. [8]	Cervical Cancer	Retrospective	KGOG-led
2021	SI Kim et al. [9]	Ovarian Cancer	Retrospective	KGOG-led
	YT Ouh et al. [10]	Cervical Cancer	Retrospective	KGOG-led
	K Kim et al. [11]	Ovarian Cancer	Retrospective	KGOG-led
2022	YJ Lee et al. [12]	Ovarian Cancer	Prospective Phase 2 Randomized	KGOG-led
	JK Sa et al. [13]	Ovarian Cancer	Retrospective	KGOG-led
	JY Lee et al. [14]	Ovarian Cancer	Prospective Phase 2 Randomized	KGOG-led
	J Park et al. [15]	Ovarian Cancer	Prospective Phase 2 Randomized	KGOG-led
	JY Park et al. [16]	Endometrial Cancer	Prospective Randomized	KGOG-led
	W Shin et al. [17]	Cervical Cancer	Retrospective	KGOG-led
2023	A. Aiob et al. [18]	Endometrial Cancer	Retrospective	KGOG-led
	SU Lee et al. [19]	Endometrial Cancer	Survey	KGOG-led
	YN Kim et al. [20]	Ovarian Cancer	Prospective Phase 2 Randomized	KGOG-led
	J Park et al. [21]	Ovarian Cancer	Prospective Phase 2 Randomized	KGOG-led
	JY Park et al. [22]	Gynecologic Cancer	Prospective Randomized	KGOG-led
2024	WY Hwang et al. [23]	Cervical Cancer	Retrospective	KGOG-led
	BS Yun et al. [24]	Cervical Cancer	Trial protocol	KGOG-led
	DH Jang et al. [25]	Endometrial Cancer	Retrospective	KGOG-led
	SI Kim et al. [26]	Ovarian Cancer	Prospective Phase 2	KGOG-led
	J Park et al. [27]	Ovarian Cancer	Prospective Phase 2	KGOG-led
	NK Kim et al. [28]	Endometrial Cancer	Retrospective	KGOG-led
	CH Choi et al. [29]	Gynecologic Cancer	Retrospective	KGOG-led

**Table 3:** Ongoing clinical trials by KGOG in 2024.

Protocol	Study Title	KGOG-Led or Participated	CRIS ID/ ClinicalTrials.gov ID
Cervix, Vulva, Vagina TSC			
KGOG 1028s	Radiotherapy versus observation in intermediate risk patients after radical hysterectomy for early cervical cancer (R-OBERV): Ancillary analysis of a Korean Gynecologic Group Study	KGOG-Led	-/-
KGOG 1036	A PROBE (prospective specimen collection and retrospective blinded evaluation) study of HPV viral load to improve cervical cancer screening	KGOG-Led	KCT0009466/ -
KGOG 1038	Phase II study of Belotecan monotherapy in patients with recurrent or persistent cervical cancer	KGOG-Led	KCT0004484/ -
KGOG 1046	AFTER trial: Adjuvant chemotherapy vs. radiotherapy for postoperative cervicalcancer—A Phase III trial	Participated	KCT0006893/ -
KGOG 1047	Therapeutic effect of surgical debulking of metastatic lymph nodes in cervical cancer Stage IIICr: A Phase III, randomized controlled clinical trial (DEBULK trial)	KGOG-Led	KCT0007137/ NCT05421650
KGOG 1049	GOG-3043: A randomized controlled trial of robotic versus open radical hysterectomy for cervical cancer (ROCC trial)	Participated	KCT0009284/ NCT04831580
Uterine Corpus-GTT TSC			
KGOG 2020	Phase II study of fertility-sparing management using high-dose oral progestin in young women with Stage I endometrial adenocarcinoma with grade 2 differentiation or superficial myometrial invasion	KGOG-Led	KCT0003226/ NCT03567655
KGOG 2024	Phase 2 trial for chemo-resistant gestational trophoblastic neoplasias with Pembrolizumab (CR-GTP)	KGOG-Led	KCT0005469/ NCT04303884
KGOG 2029	Randomized comparison between sentinel lymph node mapping and lymph node dissection in early-stage endometrial cancer	KGOG-Led	KCT0006852/ NCT04845828
KGOG 2030	Development of biomarker predicting high-dose progesterone therapy in reproductive aged women with early-stage endometrial cancer	KGOG-Led	-/-
KGOG 2031	A Phase II trial evaluating the efficacy and safety of repeated high-dose MPA therapy for patients with recurrent early-stage endometrial cancer or atypical endometrial hyperplasia	Participated	jRCTs031200256 *
KGOG 2034	Pelvic and para-aortic lymphadenectomy in patients with Stage I or II endometrial cancer with high risk of recurrence (ECLAT; AGO-OP.6); a multi-center, prospective randomized controlled trial	Participated	KCT0004981/ NCT03438474
KGOG 2035	A Phase II/III study of Paclitaxel/Carboplatin alone or combined with either Trastuzumab and Hyaluronidase-oysk (HERCEPTIN HYLECTA) or Pertuzumab, Trastuzumab, and Hyaluronidase-zzxf (PHESGO) in HER2-positive, Stage I-IV endometrial serous carcinoma or carcinosarcoma	Participated	KCT0009300/ NCT05256225
Ovary–Fallopian tube TSC			
KGOG 3033	Prospective study evaluating a strategy of surgery alone and surveillance in FIGO Stage I malignant ovarian germ cell tumor	KGOG-Led	-/-
KGOG 3050	Laparoscopic/robotic vs. open surgery in the treatment of apparently early-stage epithelial ovarian cancer	KGOG-Led	-/-
KGOG 3051	NRG-GY019: A randomized Phase III, two-arm trial of Paclitaxel/Carboplatin/maintenance Letrozole versus Letrozole monotherapy in patients with Stage II-IV, primary low-grade serous carcinoma of the ovary or peritoneum	Participated	KCT0005153/ NCT04095364
KGOG 3053	NRG CC-008 clinical trial: A non-randomized prospective clinical trail comparing the non-inferiority of salpingectomy to salpingo-oophorectomy to reduce the risk of ovarian cancer among BRCA1 carriers (SOROC)	Participated	KCT0007701/ NCT04251052
KGOG 3055	JGOG3024: Prospective cohort study of variant carriers with BRCA1 and BRCA2	Participated	-/ NCT03296826
KGOG 3058	ESPOIR: Effectiveness and safety of PARP-inhibitor therapy in recurrent ovarian cancer patients in the real world; Ambidirectional multi-center cohort study	Participated	-/-
KGOG 3059	PMFO: A cohort study to assess the safety and efficacy of PARP inhibitor maintenance after first-line therapy of Korean women with advanced ovarian/fallopian/peritoneal cancer	Participated	-/-
KGOG 3060	Effectiveness of routine intensive CA125 monitoring in ovarian cancer: A pragmatic randomized study	KGOG-Led	KCT0007454/ -
KGOG 3062	Phase III randomized clinical trial for Stage III epithelial ovarian cancer randomizing between primary cytoreductive surgery with or without hyperthermic intraperitoneal chemotherapy (OVHIPEC-2)	Participated	-/ NCT03772028
KGOG 3064	Prospective multi-institutional Phase III trial of salvage systemic therapy with or without stereotactic ablative radiation therapy for recurrent ovarian cancer (SABR-ROC)	KGOG-Led	KCT0007950/ NCT05444270
KGOG 3066	A multi-center, open-label, single-arm, Phase II study to evaluate the efficacy and safety of JPI-547, a PARP/TNKS dual inhibitor in platinum-resistant, advanced/relapsed ovarian cancer patients previously treated with a PARP inhibitor	Participated	-/ NCT05475184
KGOG 3067	A randomized Phase II study of secondary cytoreductive surgery in patients with relapsed ovarian cancer who have progressed on PARP inhibitor maintenance (SOCCER-P)	KGOG-Led	KCT0008662/ -
Surgery, Developmental Diagnostic, and Therapeutics (DDT) Committee			
KGOG 4005	The analysis of the patients who have received reoperation within 30 days after debulking operation for primary advanced epithelial ovarian, tubal, peritoneal cancer	KGOG-Led	-/-
KGOG 4006	The analysis of 90-day postoperative mortality after debulking operation for primary advanced epithelial ovarian, tubal, peritoneal cancer	KGOG-Led	-/-
KGOG 4007	Effective drug therapy for recurrent ovarian clear cell carcinoma	KGOG-Led	-/-
KGOG 4008	Study on the clinical utility of circulating tumor DNA methylation in the screening of uterine and ovarian cancers (CIRCUS)	KGOG-Led	-/-
KGOG 4009	Efficacy and safety evaluation of advanced vessel sealing device (Vi-sealer)	KGOG-Led	KCT0008008/ NCT05629611
KGOG 4010	Assessment of quality of life after low anterior resection or visceral peritoneal stripping during cytoreductive surgery for advanced ovarian cancer requiring tumor resection on the rectosigmoid colon: a prospective cohort study	KGOG-Led	-/ NCT05431530
KGOG 4011	A prospective randomized controlled trial evaluating the safety and efficacy of patient blood management program in patients with gynecologic cancer	KGOG-Led	-/ NCT05669872

* Japan Registry of Clinical Trials.

## Data Availability

Not applicable.

## Supplementary Materials

The following supporting information can be downloaded at: https://www.mdpi.com/article/10.3390/cancers16193422/s1, Figure S1: Distribution of KGOG-Participant Institutions.
