# Supplementary figures and images for: From the Beginning of the Korean Gynecologic Oncology Group to the Present and Next Steps
Source: Cancers (Basel). 2024 Oct 9;16(19):3422. doi: 10.3390/cancers16193422 (PMC11475356; doi:10.3390/cancers16193422)

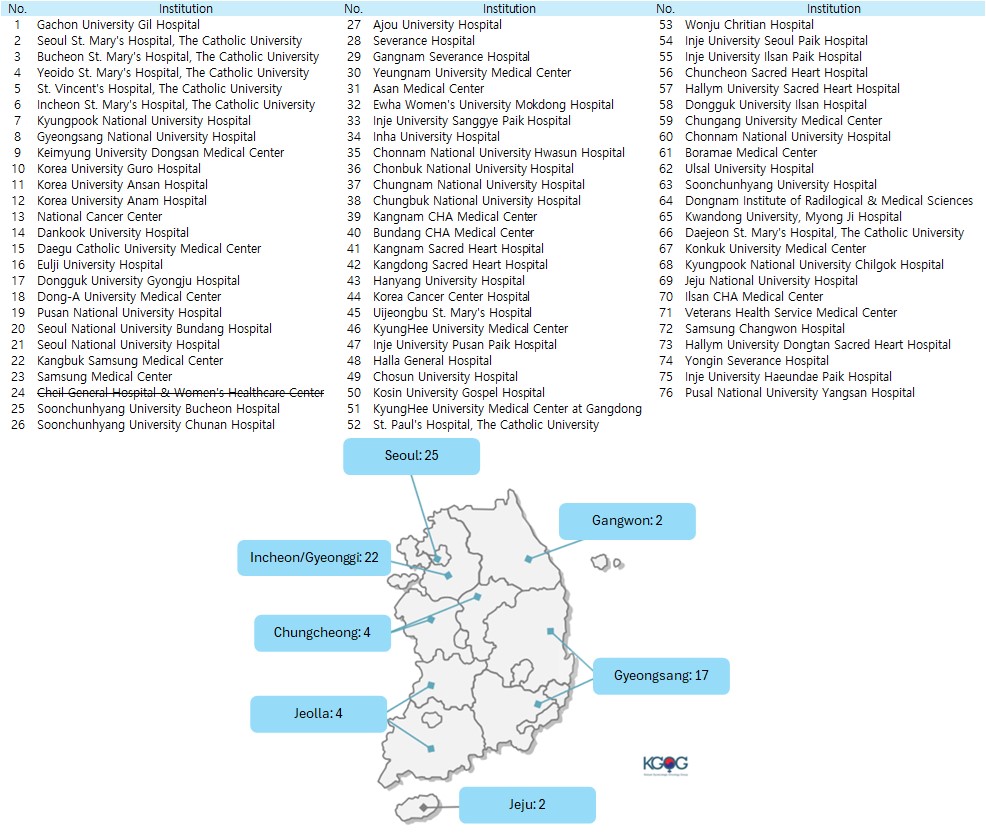

Supplement: Supplementary file 1 [file cancers-16-03422-s001.zip › cancers-3177457-supplementary.jpg]
